# Mental Health Changes in Adolescents and Adults With Cystic Fibrosis After Initiation of Elexacaftor/Tezacaftor/Ivacaftor Therapy: Insights From the Longitudinal Resilience Impacted by Positive Stressful Events (RISE) Study

**DOI:** 10.1016/j.chpulm.2025.100146

**Published:** 2025-02-07

**Authors:** Els van der Heijden, Rutger M. van den Bor, Marlou C. Bierlaagh, Danya Muilwijk, Jessica M. de Graaf, Sanne L. Nijhof, Inez Bronsveld, Cornelis K. van der Ent, Sabine E.I. van der Laan

**Affiliations:** a Department of Pediatric Pulmonology, Wilhelmina Children’s Hospital, University Medical Center Utrecht, Utrecht University, Utrecht, The Netherlands (member of ERN-LUNG); b Department of Data Science and Biostatistics, Julius Center for Health Sciences and Primary Care, University Medical Center Utrecht, Utrecht University, Utrecht, The Netherlands; c Department of Pediatrics, Wilhelmina Children’s Hospital, University Medical Center Utrecht, Utrecht University, Utrecht, The Netherlands; d Department of Pulmonary Diseases, University Medical Center Utrecht, Utrecht University, Utrecht, The Netherlands

**Keywords:** cystic fibrosis, mental health, CFTR modulator, elexacaftor/tezacaftor/ivacaftor, cohort study

## Abstract

**Background:**

Starting elexacaftor/tezacaftor/ivacaftor (ETI) is considered a positive life event for people with cystic fibrosis (pwCF). Nevertheless, some individuals report a decline in mental health after commencing ETI.

**Research Question:**

How do various mental health indicators of pwCF ≥ 12 years of age change after starting ETI, and can potentially more vulnerable subgroups, in terms of their mental health, be identified?

**Study Design and Methods:**

This was a prospective 60-week longitudinal cohort study with questionnaire-based measurements of mental health 12 weeks before (t0) and 12, 24, and 48 weeks after starting ETI (t1, t2, and t3, respectively). Subgroups were defined by age, sex, lung function at baseline, earlier use of any other modulator, and use of psychotropic medications. Data were analyzed using a covariance pattern model with a general variance covariance matrix.

**Results:**

In total, 174 (98.31%), 146 (82.49%), 141 (79.66%), and 142 (80.23%) participants completed or partially completed the questionnaires at t0, t1, t2, and t3, respectively. The data showed an overall statistically significant and clinically relevant improvement in psychosocial health (*P* < .001; change in [Δ] estimated marginal means [EMM] t0-t3, 7.68), no change in anxiety symptoms (*P* = .46; ΔEMM t0-t3 = −0.42), a statistically significant but not clinically relevant improvement in depressive symptoms (*P* < .001; ΔEMM t0-t3 = −1.25), and a statistically significant and clinically relevant improvement in respiratory-related quality of life (*P* < .001; ΔEMM t0-t3 = 19.55) after the initiation of ETI. Female patients, those with lower lung function, and those using psychotropic medications at baseline seem to be more susceptible to lower mental health scores after starting ETI on several indicators, compared with their counterparts.

**Interpretation:**

In this study, pwCF experienced an improvement in all mental health indicators, except in anxiety symptoms. Clinical physicians should be aware that female patients, people with lower lung function, and those using psychotropic medications might be more prone to less favorable mental health improvement.

**Take-Home Points**

**Study Question:** What are potential changes in various indicators of mental health among individuals with cystic fibrosis aged ≥ 12 years after the initiation of elexacaftor/tezacaftor/ivacaftor (ETI), and what differences in indicators of mental health between subgroups may identify those who are potentially more prone to a diminished mental health after starting ETI?

**Results:** The Resilience Impacted by Positive Stressful Events (RISE) study for people with cystic fibrosis demonstrated an overall statistically significant and clinically relevant improvement in psychosocial health, no change in anxiety symptoms, a statistically significant but not clinically relevant improvement in depressive symptoms, and a statistically significant and clinically relevant improvement in respiratory-related quality of life after the initiation of ETI; however, female patients, those with a lower lung function, and those using psychotropic medications at baseline seem to be more prone to lower mental health scores after starting ETI.

**Interpretation:** Clinical physicians should be aware that some subgroups might be more vulnerable to mental health changes after ETI, even though in general people with cystic fibrosis experienced improvements in most mental health indicators.

The advent of cystic fibrosis transmembrane conductance regulator (CFTR) modulators has marked a new era in the management of cystic fibrosis (CF).^1^^,^^2^ The use of the elexacaftor/tezacaftor/ivacaftor (ETI) combination significantly improves lung function and reduces pulmonary exacerbations.^2^^,^^3^ Moreover, 2 randomized, double-anonymized, placebo-controlled trials (> 12 years, N = 405,^4^ N = 113^5^) presented both a significant increase in Cystic Fibrosis Questionnaire-Revised respiratory domain (CFQ-R RD) scores^4^^,^^5^ and an improvement in almost all nonrespiratory (health-related) quality of life (QoL) domains of the Cystic Fibrosis Questionnaire-Revised (CFQ-R).^6^ For many people with cystic fibrosis (pwCF), gaining access to ETI is therefore considered a positive and major life event.^7^

Unexpectedly, several case series reported a subset of individuals who experienced a decline in their mental health after initiation of ETI.^8^, ^9^, ^10^ A review indicated that depression-related events in pwCF using ETI were generally in agreement with the baseline rate of such events in the overall CF population; therefore, this review did not imply a causal relationship with ETI.^11^ Longitudinal studies examined changes in mental health after initiation of ETI.^12^, ^13^, ^14^ Some studies identified changes in anxiety and depressive symptoms at the group level,^12^ and others reported improvements in depressive symptoms, although not in anxiety symptoms.^13^^,^^14^ Although both individuals with and without a history of mental health problems could experience a worsening of their mental health after ETI,^8^, ^9^, ^10^^,^^12^ those using psychotropic medication seemed to be more at risk for mental health changes.^10^^,^^12^^,^^15^ Furthermore, women seemed to be more prone than men to mental health problems after initiation of ETI.^13^^,^^14^ However, the aforementioned longitudinal research only included adults, and with the exception of a retrospective study,^12^ had a maximum follow-up length of 6 months.^12^, ^13^, ^14^

The Resilience Impacted by Positive Stressful Events (RISE) study for pwCF therefore aimed to investigate the potential changes in various indicators of mental health among individuals with CF aged ≥ 12 years after the initiation of ETI, with up to 48 weeks of ETI therapy. The secondary objective involved the investigation of differences in indicators of mental health between subgroups to identify those who are potentially more prone to a diminished mental health after starting ETI.

## Study Design and Methods

### Study Population and Ethics

Inclusion criteria were (1) pwCF who were aged ≥ 12 years, (2) pwCF who started ETI based on their CFTR mutation, and (3) patients at the University Medical Center Utrecht/Wilhelmina Children’s Hospital, The Netherlands. Participants were excluded when they were not able to read and understand Dutch. This study was conducted in accordance with the principles of the Declaration of Helsinki and classified by the institutional review board as exempt from the Medical Research Involving Human Subjects Act (code METC: 21/626 and 16/668).

### Study Design and Procedures

The design of the RISE study has been described in detail elsewhere.^16^ In short, this is a single-arm, observational, longitudinal cohort study which follows pwCF over a time frame of 60 weeks collecting data at 4 time points. The baseline assessment (September 2021, t0) was approximately 12 weeks before starting ETI and at 12 (April 2022, t1), 24 (August 2022, t2), and 48 weeks (November 2022, t3) after starting ETI. A package of multiple questionnaires was sent at all 4 time points. At t0 and t2, participants also attended medical evaluations.

### Indicators of Mental Health

Mental health was assessed by measuring psychosocial health, anxiety symptoms, depressive symptoms, and respiratory-related QoL using the combined emotional, social, and school/study/work subscales^17^ of the Pediatric Quality of Life Inventory (PedsQL),^18^ the Generalized Anxiety Disorder-7 (GAD-7) scale,^19^ the Patient Health Questionnaire-9 (PHQ-9),^20^ and the CFQ-R RD,^21^ respectively.

The PedsQL measures QoL and has been validated for children, adolescents, and adults.^22^ The maximum score is 100, and a minimal clinically important difference (MCID) is set at 4.4 points.^23^ The GAD-7 measures anxiety symptoms experienced in the last 2 weeks, using 7 items with a 4-point Likert scale.^19^ The maximum score is 21, the cutoff value of clinically relevant symptoms is ≥ 10, and the MCID is set at 4.0.^24^ The PHQ-9 measures depressive symptoms, experienced in the last 2 weeks, using 9 items with a 4-point Likert scale.^20^ The maximum score is 27, the cutoff value of clinically relevant symptoms is ≥ 10, and the MCID ranges from 3 to 5 points.^25^^,^^26^ The CFQ-R is a 50-item self-report questionnaire measuring health-related QoL in pwCF. The CFQ-R comprises 12 distinct domains, ranging from 0 to 100, with higher scores reflecting better QoL.^21^ We focused on the CFQ-R RD, for which the MCID was set at 4.0 points.^4^

### Clinical Study Variables

The clinical study variables at t0 and t2 were lung function (in percent predicted FEV_1_ [FEV_1_pp]), BMI, sweat chloride concentration (SCC), and fecal elastase. Moreover, by using the electronic patient files, we counted the frequency of pulmonary exacerbations (PEx) requiring IV antibiotic treatment in the year before the initial visit (t0), defined as IV-treated PEx. Additionally, we collected information on maintenance therapy including psychotropic medications. Some of the participants had used psychotropic medicines within 6 months before or at t0. The psychotropic medication used included antipsychotics, hypnotics, antidepressants, and stimulants. Unfortunately, we did not have information on mental health diagnoses of the participants. At t2, we recorded side effects in 2 ways: (1) through an open-ended question and (2) by verbally reporting a predefined list of side effects based on previous research^4^^,^^5^ and clinical knowledge, alongside any changes in the use of psychotropic medications.

### Statistical Analyses

Descriptive baseline characteristics of the cohort were presented as counts with percentages, means with SDs, or medians with interquartile ranges (IQRs). The PedsQL psychosocial health scores, GAD-7, PHQ-9, and CFQ-R scores were treated as continuous and analyzed using a covariance pattern model with a general (ie, unrestricted) variance covariance matrix. The test of primary interest was the likelihood-ratio test (α = .05) comparing the model with a fixed effect of visit against the intercept-only model, thus testing whether there was a difference in the mean score of the mental health indicator at 1 of the 4 time points. Visit-specific estimated marginal means (EMM) was provided along with 95% CIs.

To identify those potentially more vulnerable to diminished mental health after starting ETI, subgroup analyses were conducted. Subgroups were defined based on clinical practice and literature,^27^^,^^28^ and categorized into age (adolescent: < 25 years, and adult: ≥ 25 years), sex (female and male), lung function at baseline (≤ 70% and > 70%), earlier use of any other CFTR modulator (yes and no), and use of psychotropic medication at baseline (yes and no). Adolescence was defined as being aged < 25 years because this cutoff reflects the alignment with key biological developments and significant social role transitions during this time period.^29^

First, we analyzed whether we observed a difference in EMM between the subgroups by testing the full model, including visit, main effect of the subgroup, and the interaction between main effect and visit, against a model only including the effect of the visit using likelihood-ratio tests. To assess the results in more detail, we then tested whether mean differences were consistent (constant difference) and/or representing a differential change over time between the subgroups. A consistent difference was tested by using a likelihood-ratio test comparing a model including both a visit term and the main effect of the subgroup vs a model with only the visit effect. A differential change over time was analyzed by using a likelihood-ratio test comparing the full model vs the model without the interaction term. Furthermore, visit-specific contrasts were evaluated in scores between the subgroups. Finally, the physical changes in BMI, FEV_1_pp, and SCC were reported by using a covariance pattern model with a general (ie, unrestricted) variance covariance matrix.

As outlined in our study protocol, multiple imputation was to be used if a substantial portion (defined to be > 10%) of data was missing. Imputation was used by chained equations with the R package mice v3.16.0^30^; 100 data sets were imputed (using 100 iterations in each). For more information, see e-Appendix 1. Note that, whereas the results in terms of EMM were generally comparable before and after multiple imputation, *P* values for model comparisons (ie, those based on the pooled [D2] likelihood ratio test statistic) displayed substantial variability. Exact causes of this variability remained unclear; however, the variability may be related to the specific pooling procedure that has been the focus of criticism in the methodologic literature.^31^ It was therefore decided to present the results of the original data. For comparison between results based on multiply imputed data and original data, see e-Table 1.

Significance levels were set at 0.05. No correction was done for multiple testing. All statistical analyses were performed with R version 4.4.1 (R core team, Austria); besides the mice package previously cited, we used the packages miceadds v3.17-44,^32^ nlme v3.1-166,^33^ and package emmeans v1.10.4.^34^

## Results

### Study Population

In total, 177 patients participated in the study, with a mean age of 25.80 years. Of these, 44.07% were female, and 79.10% had a F508del/F508del mutation (Table 1). In total 174 (98.31%), 146 (82.49%), 141 (79.66%), and 142 (80.23%) participants completed or partially completed the questionnaires at t0, t1, t2, and t3, respectively. Altogether, 66.10% of the participants completed the RISE questionnaires at all 4 time points, 84.70% at least at 3 time points, and 90.30% at least at 2 time points. Not all questionnaires were 100% completed (e-Table 2). Seven participants dropped out of the study cohort: 3 without an explicit explanation, 1 was admitted to the psychiatric ICU (unclear whether ETI was continued or not), 2 discontinued ETI (1 due to liver problems, for the other no information was available on why they quit), and 1 participant was transferred to another medical center and was lost to follow-up. (The participants admitted to the psychiatric ICU and those who discontinued ETI accidentally dropped out of the study; these participants should have remained enrolled.)

**Table 1:** Baseline Characteristics of Study Population (N = 177)

Participant Characteristics and Medical Information	Value
Age at baseline	25.80 [8.79]
Adolescents: 12-24 y	94 (53.11)
Adults: ≥ 25 y	83 (46.89)
Male	99 (55.93)
Female	78 (44.07)
Genotype	
F508del/F508del	140 (79.10)
F508del/any other mutation	37 (20.90)
BMI, kg/m^2^	21.12 [2.68]
FEV_1_pp	73.18 [19.62]
< 40	6 (3.39)
40-70	67 (37.85)
70-90	64 (36.16)
> 90	40 (22.60)
SCC, mmol·L^−1^	84.27 [17.32]
Missing^a^	6 (3.39)
Pancreatic function	
Insufficient, fecal elastase < 200 μg·g^−1^	157 (88.70)
Sufficient, fecal elastase ≥ 200 μg·g^−1^	3 (1.69)
Missing	17 (9.60)
CFRD	53 (29.94)
Use of CFTR modulator before ETI	
Yes	141 (79.66)
No	36 (20.34)
IV-treated PExs (before t0)	
None	137 (77.40)
≥ 1	40 (22.60)
Use of psychotropic medication	
Yes	16 (9.04)
No	161 (90.96)
Questionnaires	
Completed^b^	174 (98.31)
Psychosocial health (PedsQL^c^; 0-100) (n = 173)	72.48 [14.73]
Anxiety symptoms (GAD-7; 0-21) (n = 170)	3.59 [3.30]
Depressive symptoms (PHQ-9; 0-27) (n = 170)	5.09 [4.06]
Respiratory-related QoL (CFQ-R RD; 0-100) (n = 163)	68.81 [20.87]

Data are presented as n (%), or mean [SD]. CFQ-R RD = Cystic Fibrosis Questionnaire-Revised respiratory domain; CFRD = cystic fibrosis-related diabetes; ETI = elexacaftor/tezacaftor/ivacaftor; FEV_1_pp = FEV_1_ percent predicted; GAD-7 = Generalized Anxiety Disorder-7; PedsQL = Pediatric Quality of Life Inventory 4.0; PEx = pulmonary exacerbation; PHQ-9 = Patient Health Questionnaire-9; QoL = quality of life; SCC = sweat chloride concentration. Original data is used.

a Insufficient sweat collection.

b Not all questionnaires 100% completed.

c We used validated versions adolescents and adults.

### Changes in Mental Health Indicators After Initiation of ETI

**Figure 1:**
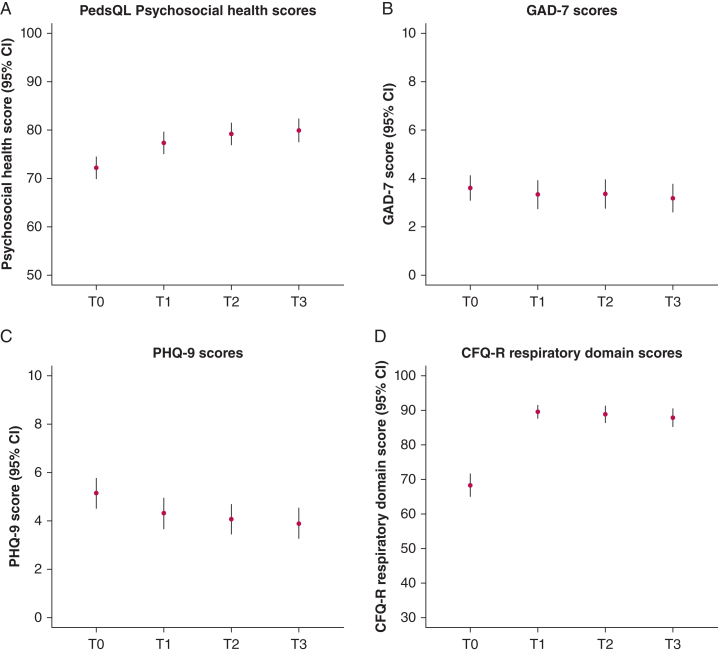
A-D, Estimated marginal means with 95% CIs at t0, t1, t2, and t3 with (A) PedsQL psychosocial health scores, (B) GAD-7 scores, (C) PHQ-9 scores, and (D) CFQ-R respiratory domain scores. CFQ-R = Cystic Fibrosis Questionnaire-Revised; GAD-7 = Generalized Anxiety Disorder-7; PedsQL = Pediatric Quality of Life Inventory; PHQ-9 = Patient Health Questionnaire-9. Original data are used.

All results are presented in Table 2 and visualized in Figure 1.

**Table 2:** Changes in Mental Health Indicators on Group Level and Subgroup Level

Outcome	t0	t1	t2	t3	*P* Values			
	EMM (95% CI)	EMM (95% CI)	EMM (95% CI)	EMM (95% CI)	Overall	Post Hoc Tests		
						t0-t1	t1-t2	t2-t3
Psychosocial health scores (0-100)	72.24 (70.02-74.46)	77.41 (75.16-79.67)	79.26 (77.04-81.47)	79.92 (77.61-82.24)	< .001	< .001	.005	.25

Subgroups Analyses	EMM (95% CI)	EMM (95% CI)	EMM (95% CI)	EMM (95% CI)	Difference in Visits^a^	Post Hoc Tests		
						Consistent Difference^b^	Differential Change^c^	
Age at t0								
< 25 y	74.49 (71.47-77.51)	78.94 (75.75-82.13)	81.72 (78.82-84.63)	82.15 (79.12-85.19)	.20	.12	.50	
≥ 25 y	69.79 (66.52-73.05)	75.78 (72.61-78.94)	76.63 (73.32-79.95)	77.46 (73.95-80.97)				
Sex								
Female	70.47 (67.09-73.85)	76.14 (72.82-79.46)	77.18 (73.78-80.58)	78.24 (74.43-82.06)	.50	.29	.76	
Male	73.65 (70.69-76.61)	78.47 (75.41-81.53)	80.86 (77.94-83.79)	81.27 (78.35-84.19)				
Lung function at t0								
FEV_1_pp ≤ 70	68.85 (65.66-72.04)	76.20 (72.80-79.59)	77.41 (73.86-80.96)	77.90 (74.02-81.77)	.008	.02	.08	
FEV_1_pp > 70	74.96 (71.98-77.95)	78.28 (75.23-81.33)	80.75 (77.93-83.57)	81.66 (78.89-84.44)				
Earlier use of CFTR modulator								
Yes	72.95 (70.49-75.41)	77.96 (75.48-80.43)	80.02 (77.55-82.49)	80.30 (77.74-82.86)	.76	.67	.62	
No	69.57 (64.39-74.74)	75.37 (69.92-80.83)	76.33 (71.36-81.30)	78.52 (73.05-83.99)				
Use psychotropic medications at t0								
Yes	62.62 (55.34-69.90)	66.15 (57.10-75.20)	69.67 (61.27-78.06)	66.90 (58.00-75.80)	.04	.02	.36	
No	73.23 (70.93-75.52)	78.51 (76.23-80.79)	80.16 (77.89-82.43)	81.20 (78.86-83.53)				

Outcome	t0	t1	t2	t3	*P* Values			
	EMM (95% CI)	EMM (95% CI)	EMM (95% CI)	EMM (95% CI)	Overall	Post Hoc Tests		
						t0-t1	t1-t2	t2-t3
Anxiety symptoms (0-21)	3.61 (3.11-4.11)	3.34 (2.77-3.91)	3.36 (2.78-3.94)	3.19 (2.63-3.76)	.46	.31	.94	.43


Subgroup Analyses	EMM (95% CI)	EMM (95% CI)	EMM (95% CI)	EMM (95% CI)	Difference in Visits^a^	Post Hoc Tests		
						Consistent Difference^b^	Differential Change^c^	
Age at t0								
< 25 y	3.21 (2.57-3.85)	3.48 (2.65-4.31)	3.06 (2.28-3.83)	3.29 (2.47-4.11)	.08	.28	.049	
≥ 25 y	4.04 (3.27-4.82)	3.18 (2.41-3.95)	3.66 (2.79-4.53)	3.11 (2.31-3.91)				
Sex								
Female	4.00 (3.21-4.79)	3.02 (2.23-3.81)	3.72 (2.74-4.71)	3.55 (2.60-4.49)	.01	.06	.03	
Male	3.31 (2.67-3.95)	3.55 (2.76-4.35)	3.13 (2.41-3.85)	2.96 (2.24-3.67)				
Lung function at t0								
FEV_1_pp ≤ 70	3.90 (3.19-4.61)	3.67 (2.83-4.52)	3.97 (3.06-4.87)	3.34 (2.53-4.16)	.21	.31	.18	
FEV_1_pp > 70	3.37 (2.67-4.07)	3.06 (2.29-3.84)	2.85 (2.11-3.59)	3.12 (2.31-3.94)				
Earlier use of CFTR modulator								
Yes	3.40 (2.82-3.98)	3.10 (2.47-3.74)	3.16 (2.54-3.79)	2.91 (2.28-3.53)	.23	.09	.82	
No	4.42 (3.45-5.38)	4.27 (3.00-5.54)	4.02 (2.59-5.45)	4.28 (2.99-5.58)				
Use psychotropic medications at t0								
Yes	4.28 (3.20-5.36)	5.32 (2.94-7.69)	3.84 (1.95-5.74)	4.44 (2.59-6.29)	.02	.01	.25	
No	3.54 (3.00-4.08)	3.15 (2.58-3.73)	3.32 (2.71-3.94)	3.08 (2.48-3.68)				


Outcome	t0	t1	t2	t3	*P* Values			
	EMM (95% CI)	EMM (95% CI)	EMM (95% CI)	EMM (95% CI)	Overall	Post Hoc Tests		
						t0-t1	t1-t2	t2-t3
Depressive symptoms (0-27)	5.14 (4.52-5.75)	4.31 (3.67-4.95)	4.06 (3.46-4.66)	3.89 (3.28-4.50)	< .001	.003	.28	.41


Subgroup Analyses	EMM (95% CI)	EMM (95% CI)	EMM (95% CI)	EMM (95% CI)	Difference in Visits^a^	Post Hoc Tests		
						Consistent Difference^b^	Differential Change^c^	
Age at t0								
< 25 y	4.66 (3.86-5.47)	4.38 (3.48-5.28)	4.04 (3.28-4.80)	3.83 (2.97-4.69)	.11	.16	.17	
≥ 25 y	5.65 (4.69-6.61)	4.21 (3.31-5.12)	4.07 (3.13-5.01)	3.96 (3.08-4.84)				
Sex								
Female	5.56 (4.48-6.63)	4.60 (3.65-5.55)	4.52 (3.56-5.49)	4.38 (3.33-5.42)	.14	.04	.94	
Male	4.80 (4.06-5.55)	4.04 (3.19-4.89)	3.70 (2.93-4.46)	3.53 (2.78-4.28)				
Lung function at t0								
FEV_1_pp ≤ 70	5.97 (5.03-6.91)	4.77 (3.82-5.73)	4.53 (3.57-5.49)	3.95 (3.04-4.85)	.06	.33	.03	
FEV_1_pp > 70	4.46 (3.65-5.27)	3.93 (3.07-4.79)	3.68 (2.92-4.43)	3.91 (3.07-4.74)				
Earlier use of CFTR modulator								
Yes	4.97 (4.24-5.69)	4.18 (3.47-4.88)	3.89 (3.21-4.58)	3.83 (3.17-4.48)	.24	.10	.75	
No	5.77 (4.57-6.97)	4.78 (3.28-6.29)	4.72 (3.47-5.97)	4.11 (2.58-5.63)				
Use psychotropic medications at t0								
Yes	7.22 (4.95-9.50)	6.32 (3.71-8.93)	6.40 (4.27-8.52)	5.86 (3.90-7.83)	.38	.15	.94	
No	4.94 (4.30-5.58)	4.11 (3.46-4.76)	3.83 (3.21-4.45)	3.71 (3.07-4.35)				


Outcome	t0	t1	t2	t3	*P* Values			
	EMM (95% CI)	EMM (95% CI)	EMM (95% CI)	EMM (95% CI)	Overall	Post Hoc Tests		
						t0-t1	t1-t2	t2-t3
Respiratory-related QoL (0-100)	68.28 (64.99-71.56)	89.47 (87.69-91.26)	88.77 (86.44-91.10)	87.83 (85.37-90.29)	< .001	< .001	.56	.38


Subgroup Analyses	EMM (95% CI)	EMM (95% CI)	EMM (95% CI)	EMM (95% CI)	Difference in Visits^a^	Post Hoc Tests		
						Consistent Difference^b^	Differential Change^c^	
Age at t0								
< 25 y	71.37 (66.85-75.90)	89.02 (86.47-91.57)	88.89 (85.96-91.82)	88.21 (85.06-91.37)	.12	.20	.14	
≥ 25 y	64.90 (60.17-69.64)	89.94 (87.39-92.49)	88.55 (84.86-92.24)	87.42 (83.56-91.21)				
Sex								
Female	64.38 (58.98-69.79)	87.84 (84.70-90.99)	87.42 (83.32-91.52)	88.08 (84.03-92.12)	.01	.01	.18	
Male	71.35 (67.30-75.41)	90.64 (88.52-92.75)	89.89 (87.19-92.59)	87.72 (84.53-90.90)				
Lung function at t0								
FEV_1_pp ≤ 70	59.46 (54.63-64.30)	88.04 (85.42-90.66)	87.20 (83.64-90.75)	86.77 (83.45-90.09)	< .001	.09	< .001	
FEV_1_pp > 70	75.93 (72.04-79.82)	90.65 (88.19-93.11)	89.92 (86.80-93.04)	88.63 (85.04-92.22)				
Earlier use of CFTR modulator								
Yes	70.65 (67.09-74.22)	89.68 (87.56-91.80)	88.92 (86.32-91.52)	87.75 (84.86-90.64)	.009	.06	.02	
No	58.40 (50.48-66.32)	88.72 (85.65-91.80)	88.36 (82.85-93.87)	88.17 (83.89-92.44)				
Use psychotropic medications at t0								
Yes	57.49 (45.98-68.99)	88.54 (83.44-93.64)	83.20 (76.31-90.09)	76.39 (65.48-87.30)	.01	.03	.06	
No	69.36 (65.96-72.76)	89.50 (87.58-91.43)	89.25 (88.75-91.75)	88.96 (86.56-91.36)				

CFTR = cystic fibrosis transmembrane conductance regulator; EMM = estimated marginal means; QoL = quality of life. Original data is used for this table.

a A low *P* value suggests that there are likely differences in the subgroups, rather than assuming that there is no difference between subgroups nor between visits.

b A low *P* value suggests that it is more likely there is a consistent difference in the subgroups means across visits, rather than assuming that the subgroups are the same at each visit.

c A low *P* value suggests that it is more likely there are changing differences in subgroup means, rather than assuming the difference is always the same.

### Psychosocial Health

The EMMs of the PedsQL psychosocial health scores were 72.24 (95% CI, 70.02-74.46) at t0, 77.41 (95% CI, 75.16-79.67) at t1, 79.26 (95% CI, 77.04-81.47) at t2, and 79.92 (95% CI, 77.61-82.24) at t3, showing a significant and clinically relevant increase in scores between the 4 time points (*P* < .001; change (Δ) in EMM t0-t3 = 7.68). Post hoc tests identified that this increase is between t0 and t1 (*P* < .001), and between t1 and t2 (*P* = .005).

#### Anxiety Symptoms

The EMMs of the GAD-7 scores were 3.61 (95% CI, 3.11-4.11) at t0, 3.34 (95% CI, 2.77-3.91) at t1, 3.36 (95% CI, 2.78-3.94) at t2, and 3.19 (95% CI, 2.62-3.76) at t3. No significant or clinically relevant differences were found between the 4 time points (*P* = .46; ΔEMM t0-t3 = −0.42), nor between the specific visits.

#### Depressive Symptoms

The EMMs of the PHQ-9 scores were 5.14 (95% CI, 4.52-5.75) at t0, 4.31 (95% CI, 3.67-4.95) at t1, 4.06 (95% CI, 3.46-4.66) at t2, and 3.89 (95% CI, 3.28-4.50) at t3, showing a significant but not clinically relevant decrease in depressive symptoms between the 4 time points (*P* < .001; ΔEMM t0-t3 = −1.25). Post hoc tests identified that this change is only significant between t0 and t1 (*P* = .003).

#### Respiratory-Related QoL

The EMMs of the CFQ-R RD scores were 68.28 (95% CI, 64.99-71.56) at t0, 89.47 (95% CI, 87.69-91.26) at t1, 88.77 (95% CI, 86.44-91.10) at t2, and 87.83 (95% CI, 85.37-90.29) at t3, showing a significant and clinically relevant improvement in respiratory-related QoL between the 4 time points (*P* < .001; ΔEMM t0-t3 = 19.55). Post hoc tests identified that this change is only significant between t0 and t1 (*P* < .001).

### Subgroup Analyses

Only significant differences in the subgroup analyses are subsequently presented; however, all results are presented in Table 2 and visualized in e-Figure 1. Visit-specific contrasts per subgroup are reported in e-Table 3.

#### Psychosocial Health

PedsQL psychosocial health scores differed between those with a baseline FEV_1_pp ≤ 70 (ΔEMM t0-t3 = 9.05) and FEV_1_pp > 70 (ΔEMM t0-t3 = 6.70; *P* = .008). Moreover, consistent differences in scores between the subgroups (*P* = .02) suggest that, overall, a higher average PedsQL psychosocial health score is to be expected for patients with a baseline FEV_1_pp > 70 compared with those with an FEV_1_pp ≤ 70. There was however no significant differential change over time (*P* = .08).

Additionally, PedsQL psychosocial health scores differed between those who used psychotropic medications (ΔEMM t0-t3 = 4.28) and those who did not (ΔEMM t0-t3= 7.97; *P* = .04). Furthermore, those who used psychotropic medications at baseline had consistently lower PedsQL psychosocial health scores compared with those that did not use these medications (*P* = .02). However, there was no significant differential change in PedsQL psychosocial health scores over time (*P* = .36) (Table 2).

#### Anxiety Symptoms

GAD-7 scores differed between male patients (ΔEMM t0-t3= −0.05) and female patients (ΔEMM t0-t3 = −0.35; *P* = .01). No significant consistent difference in GAD-7 scores was observed between the subgroups (*P* = .06). However, there was a differential change in GAD-7 scores over time (*P* = .03), suggesting that the average GAD-7 score was higher for female patients than for male patients at t0, t2 and t3, but lower at t1.

Additionally, GAD-7 scores differed between those who used psychotropic medications (ΔEMM t0-t3 = 0.16) and those who did not (ΔEMM t0-t3 = −0.46; *P* = .02): those on psychotropic medications at baseline had consistently higher GAD-7 scores compared with those that did not use these medications (*P* = .01). There was no significant differential change over time (*P* = .25).

#### Depressive Symptoms

Overall, PHQ-9 scores did not differ in any of the subgroups. However, PHQ-9 scores consistently differed between male patients and female patients (*P* = .04), suggesting that overall a higher average PHQ-9 score is expected for female compared with male patients. Moreover, PHQ-9 scores for the subgroup based on lung function had a differential change (*P* = .03), suggesting that patients with an FEV_1_pp ≤ 70 are expected to have a greater decrease in PHQ-9 scores compared with those with an FEV_1_pp > 70.

#### Respiratory-Related QoL

CFQ-R RD scores differed in subgroups defined by sex (female ΔEMM t0-t3 = 23.70; male ΔEMM t0-t3= 16.37; *P* = .01), lung function (FEV_1_pp ≤ 70 ΔEMM t0-t3 = 27.33; FEV_1_pp > 70 ΔEMM t0-t3 = 12.70; *P* ≤ .001), earlier use of a CFTR modulator (use ΔEMM t0-t3 = 17.10; no use ΔEMM t0-t3 = 29.77; *P* = .009), and use of psychotropic medications (use ΔEMM t0-t3 = 18.90; no use ΔEMM t0-t3, 19.60 = *P* = .01). CFQ-R RD scores differed in a consistent manner for sex and use of psychotropic medications, suggesting that overall a higher CFQ-R RD score is expected for male patients (*P* = .01) and those who did not use psychotropic medications at baseline (*P* = .03) compared with female patients and those not using psychotropic medications at baseline. Differential changes in CFQ-R RD scores were found for the subgroups defined by lung function (*P* < .001) and earlier use of CFTR modulator (*P* = .02), suggesting that patients with an FEV_1_pp ≤ 70 and patients who did not use a CFTR modulator before are expected to have a greater increase in CFQ-R RD scores compared with those with an FEV_1_pp > 70 and those already using a CFTR modulator.

### Clinical Changes After ETI

Regarding clinical changes after ETI, expressed as EMM, BMI increased by 0.67 kg/m^2^ (*P* < .0001; 95% CI, 0.48-0.85), FEV_1_pp increased by 13.10% (*P* < .0001; 95% CI, 11.39-14.81), and SCC decreased by 49.34 mmol/L (*P* < .0001; 95% CI, −53.03 to −45.64) at t2 (Table 3). For more clinical changes, including experienced (mental health-related) side effects after ETI, please refer to e-Appendix 2.

**Table 3:** Clinical Changes After Initiation of Elexacaftor/Tezacaftor/Ivacaftor

Clinical Parameters	t0, EMM	t2, EMM	Difference	95% CI	*P* Value
BMI, kg/m^2^	21.12	21.79	0.67	0.48 to 0.85	< .0001
FEV_1_pp	73.18	86.27	13.10	11.39 to 14.81	< .0001
SCC, mmol/L	84.27	34.93	−49.34	− 53.03 to −45.64	< .0001

EMM = estimated marginal mean; FEV_1_pp = percent predicted FEV_1_ percent predicted; SCC = sweat chloride concentration. Original data is used.

## Discussion

The RISE study demonstrated an overall statistically significant and clinically relevant improvement in psychosocial health, no change in anxiety symptoms, a statistically significant but not clinically relevant improvement in depressive symptoms, and a statistically significant and clinically relevant improvement in respiratory-related QoL after the initiation of ETI. Notably, GAD-7 and PHQ-9 scores were low at all 4 time points, indicating a consistently reported low level of anxiety and depressive symptoms both before and after ETI use. The most substantial changes in mental health indicators occurred between 3 months before and 3 months after starting of ETI. Depending on the mental health indicator, certain subgroups appeared to demonstrate a higher susceptibility to decreased mental health after ETI initiation, as indicated by differences in mental health scores between subgroups. These differences in subgroups could be attributed either to a generally lower level of mental health (significant consistent difference) or to a different pattern of change after ETI initiation (significant differential change). Based on significant differences in ≥ 2 mental health indicators (out of the 4 measured), we can cautiously conclude that female patients, those with a FEV_1_pp ≤ 70, and those using psychotropic medications at baseline seem to be more susceptible to lower mental health scores than their counterparts.

Our findings are in agreement with multiple existing studies: they also showed no change in anxiety symptoms,^12^, ^13^, ^14^ but a statistically significant improvement in depressive symptoms,^13^^,^^14^ and a statistically significant and clinically relevant improvement in respiratory-related QoL.^4^^,^^5^^,^^35^ Previous research did not focus on changes in psychosocial health after use of ETI; therefore, no comparisons can be made. Previous literature has shown that older age, being female, and having a lower FEV_1_pp are associated with lower levels of mental health in adults in general.^36^ Depending on the mental health indicator, we also found that these subgroups might be at risk for a poorer mental health in the studied pwCF. Studies have indicated that individuals with a medical history of psychiatric illness or use of psychotropic medications were more likely to experience a worsening of their mental health after ETI.^8^^,^^10^^,^^12^^,^^15^ In our study, individuals using psychotropic medications at baseline appeared to exhibit poorer psychosocial health, heightened anxiety symptoms, and diminished respiratory-related QoL. We did, however, not find a differential change over time, suggesting that lower mental health scores appear to be independent of ETI use. These results contrast with the hypothesis that a decline in mental health observed in people using psychotropic medications may be due to a drug-drug interaction between the CFTR modulator and psychotropic medications.^9^^,^^37^ Moreover, it is known that certain individuals require changes in their psychotropic and/or alterations in CFTR medication to improve their psychiatric symptoms after ETI initiation.^8^^,^^10^^,^^12^^,^^15^ Future studies are needed to confirm these results and find underlying mechanisms at the individual level to better understand why individuals react differently to ETI in terms of their mental health. Besides, some of our results are not statistically significant, but do exceed the MCID. In our view, such study results are to be considered inconclusive, and overemphasis may be misleading. Nonetheless, these results may indicate a potentially clinically relevant change and could therefore be relevant for future studies.

The strengths of this study are the longitudinal prospective design with 4 time points of data collection, including a baseline assessment before the initiation of ETI and a long follow-up period, and the measuring of multiple indicators of mental health. The study participants included both male and female patients, adolescents and adults, and people with varying degrees of disease severity, which enhances the generalizability of the findings. Some limitations deserve mentioning. We noticed that due to the length of the questionnaires, some adolescents lacked the motivation and/or concentration to complete them during the visits. Furthermore, we ranked our key outcome variable the highest to improve data completeness, and we administered questionnaires before physical testing to prevent answers from being influenced by their physical test results. Parents might have helped to complete the questionnaires, which could have influenced the results. We expected missing data to be rare,^16^ but the response rate was lower than anticipated. Comparison of baseline distributions on age, lung function, PedsQL, PHQ-9, CFQ-R RD, and GAD-7 scores between patients who did and did not complete the RISE surveys at t3 indicated potential differences in CFQ-R RD and GAD-7 (e-Table 4). We aimed to mitigate potential bias by using multiple imputation techniques. The EMMs estimated using the imputed data were similar to the EMMs estimated without imputation; therefore, these data suggest limited attrition bias. Moreover, we cannot establish a causal relationship between ETI and psychosocial health because external factors or unrelated other life events could potentially influence our results. Furthermore, no formal correction for multiple correction was applied, and results should be interpreted accordingly. Additionally, no patients with oxygen therapy were included in the RISE study. It is likely that the most severely ill pwCF might have been on a compassionate use of ETI program, and therefore, have not been included in this study: the exact number of these participants is unavailable. Finally, although our results indicated that (most of our) outcomes improved on a group level, individual participants might have experienced side effects that could have influenced their mental health. Future research might therefore focus on examining the relationship between side effects and changes in mental health at the individual level.

## Interpretation

The RISE study underlines a clinically relevant improvement in psychosocial health, no changes in anxiety symptoms, an improvement in depressive symptoms, and a clinically relevant improvement in respiratory-related QoL after the initiation of ETI. At the subgroup level, we can cautiously conclude that female patients, those with a FEV_1_pp ≤ 70, and those using psychotropic medications at baseline might be more prone to a less favorable mental health improvement.

## Funding/Support

This study was funded by Corno Fonds Onderzoek Subsidie 2022 (CFOS) of the 10.13039/100000897Dutch Cystic Fibrosis Foundation [Grant R6266] to S. E. I. v. d. L., E. v. d. H., S. L. N., and C. K. v. d. E.

## Financial/Nonfinancial Disclosures

None declared.

## Supplementary Data

e-Online Data

e-Online Data

e-Online Data

e-Online Data

e-Online Data

e-Online Data

e-Online Data
