# Supplementary material for: Mental Health Changes in Adolescents and Adults With Cystic Fibrosis After Initiation of Elexacaftor/Tezacaftor/Ivacaftor Therapy: Insights From the Longitudinal Resilience Impacted by Positive Stressful Events (RISE) Study
Source: CHEST Pulm. 2025 Feb 7;3(3):100146. doi: 10.1016/j.chpulm.2025.100146 (PMC13418347; doi:10.1016/j.chpulm.2025.100146)
Supplement: e-Online Data [file mmc1.pdf]

Supplement 1 – More information about the imputation model

Imputations were performed with the data in wide format. Corresponding to the default options, imputations were based on predictive mean matching (numeric variables) or logistic regression (binary variables). However, using the default predictor matrix (i.e. using all variables as predictors) resulted in numerical issues for one of the variables with missing data (sweat chloride at t2). Therefore, the quickpred() function (with default specifications) was used to simplify the model for this specific variable. No issues were logged in the function's event log, and no abnormalities were identified during the inspection of trace plots. Rubin's rules were used to pool estimates from the imputed data sets. Likelihood ratio test statistics were pooled using the 'D2' method (using the function micombine.chisquare() from the R package miceadds v3.17-44 [1]. Pooled confidence intervals for estimated marginal means are based on normal approximations.

List of variables included in the imputation model; variables are treated as numeric unless indicated otherwise

t0_ApprAgeAtBaseline	
t0_pat_sex	Binary
t0_CFTR_modulator	Binary
t0_genotype_Cat	Binary
t0_BMIAtBaseline	
t0_baseline_sweatchloride	
t0_baseline_FEV1_pp	
t0_baseline_fecal_elastase	Binary (Dichotomized: 0 vs >0)<br>*fecal elastase at t2 was not included, because only one observation exceeded 0.
t0_total_dur_IV_AB	Binary (Dichotomized: 0 vs >0)
t0_clin_CFRD	Binary
t0_clin_CFLD	Binary
t0_baseline_psychfarm	Binary
t0_PedsQL_PsychSoc_total	
t0_BIPQ_total	
t0_IIQ_Rejection	
t0_IIQ_Acceptance	
t0_IIQ_Engulfment	
t0_IIQ_Enrichment	
t0_PSS_total	
t0_GAD7_total	
t0_PHQ9_total	
t0_RCADS_Overall_total	
t0_Cantril	
t0_CFQR_Resp	
t0_BRS_total	
t0_SDQ_Difficulties_total	
t1_PedsQL_PsychSoc_total	
t1_BIPQ_total	
t1_IIQ_Rejection	
t1_IIQ_Acceptance	

t1_IIQ_Engulfment	
t1_IIQ_Enrichment	
t1_PSS_total	
t1_GAD7_total	
t1_PHQ9_total	
t1_RCADS_Overall_total	
t1_Cantril	
t1_CFQR_Resp	
t1_BRS_total	
t1_SDQ_Difficulties_total	
t2_BMIAtFollowup	
t2_followup_sweatchloride	
t2_followup_FEV1_pp	
t2_sum_unresolved_sideeffects	
t2_followup_psychfarm	Binary
t2_PedsQL_PsychSoc_total	
t2_BIPQ_total	
t2_IIQ_Rejection	
t2_IIQ_Acceptance	
t2_IIQ_Engulfment	
t2_IIQ_Enrichment	
t2_PSS_total	
t2_GAD7_total	
t2_PHQ9_total	
t2_RCADS_Overall_total	
t2_Cantril	
t2_CFQR_Resp	
t2_BRS_total	
t2_SDQ_Difficulties_total	
t3_PedsQL_PsychSoc_total	
t3_BIPQ_total	
t3_IIQ_Rejection	
t3_IIQ_Acceptance	
t3_IIQ_Engulfment	
t3_IIQ_Enrichment	
t3_PSS_total	
t3_GAD7_total	
t3_PHQ9_total	
t3_RCADS_Overall_total	
t3_Cantril	
t3_CFQR_Resp	
t3_BRS_total	
t3_SDQ_Difficulties_total	

1. Robitzsch A, Grund S. miceadds: Some Additional Multiple Imputation Functions, Especially for “mice.” https://CRAN.R-project.org/package=miceadds. Accessed May 1, 2023. https://CRAN.R-project.org/package=miceadds
