# Supplementary material for: Mental Health Changes in Adolescents and Adults With Cystic Fibrosis After Initiation of Elexacaftor/Tezacaftor/Ivacaftor Therapy: Insights From the Longitudinal Resilience Impacted by Positive Stressful Events (RISE) Study
Source: CHEST Pulm. 2025 Feb 7;3(3):100146. doi: 10.1016/j.chpulm.2025.100146 (PMC13418347; doi:10.1016/j.chpulm.2025.100146)
Supplement: e-Online Data [file mmc2.pdf]

Supplement 2 – results based on original data and multiple imputed (MI) data sets

We show the results based on original and MI data sets of 'Table 2'in the main text (page 1-12 in this document), 'Supplement 5' (page 13-16 in this document), and 'Table 3' in the main text (page 17 in this document).

'Table 2' in main text

	T0				T1			
	Original		MI		Original		MI	
**Outcome**	**EMM**	**(95% CI)**	**EMM**	**(95% CI)**	**EMM**	**(95% CI)**	**EMM**	**(95% CI)**
psychosocial health [0-100]	72.24	(70.02;74.46)	72.28	(70.09;74.47)	77.41	(75.16;79.67)	77.28	(75.01;79.55)
**Subgroups analyses**	**EMM**	**(95% CI)**	**EMM**	**(95% CI)**	**EMM**	**(95% CI)**	**EMM**	**(95% CI)**
Age at T0								
<25 years	74.49	(71.47;77.51)	74.40	(71.41;77.40)	78.94	(75.75;82.13)	78.48	(75.24;81.71)
≥25 years	69.79	(66.52;73.05)	69.87	(66.72;73.02)	75.78	(72.61;78.94)	75.93	(72.80;79.06)
Sex								
Female	70.47	(67.09;73.85)	70.55	(67.22;73.88)	76.14	(72.82;79.46)	76.39	(72.99;79.78)
Male	73.65	(70.69;76.61)	73.64	(70.74;76.54)	78.47	(75.41;81.53)	77.99	(74.96;81.01)
Lung function at T0								
≤70	68.85	(65.66;72.04)	68.95	(65.90;72.00)	76.20	(72.80;79.59)	75.99	(72.70;79.28)
>70	74.96	(71.98;77.95)	74.96	(71.97;77.95)	78.28	(75.23;81.33)	78.32	(75.25;81.40)
Earlier use of CFTR modulator								
Yes	72.95	(70.49;75.41)	73.03	(70.60;75.45)	77.96	(75.48;80.43)	77.70	(75.18;80.21)
No	69.57	(64.39;74.74)	69.35	(64.39;74.32)	75.37	(69.92;80.83)	75.54	(70.35;80.74)
Use psychotropic med T0								
Yes	62.62	(55.34;69.90)	62.60	(55.84;69.36)	66.15	(57.10;75.20)	67.37	(59.32;75.43)
No	73.23	(70.93;75.52)	73.24	(70.98;75.50)	78.51	(76.23;80.79)	78.27	(75.95;80.58)

continued	T2		T3		p-values	
	**Original**	**MI**	**Original**	**MI**	**Original**	**MI**

Outcome	EMM	(95% CI)	EMM	(95% CI)	EMM	(95% CI)	EMM	(95% CI)	overall	overall
psychosocial health [0-100]	79.26	(77.04;81.47)	78.59	(76.34;80.83)	79.92	(77.61;82.24)	79.84	(77.50;82.18)	<0.0001	<0.0001
**Subgroups analyses**	**EMM**	**(95% CI)**	**EMM**	**(95% CI)**	**EMM**	**(95% CI)**	**EMM**	**(95% CI)**	**Difference in visits**	
									**Original**	**MI**
Age at T0									0.20	0.50
<25 years	81.72	(78.82;84.63)	80.59	(77.53;83.64)	82.15	(79.12;85.19)	81.64	(78.46;84.81)		
≥25 years	76.63	(73.32;79.95)	76.32	(73.11;79.53)	77.46	(73.95;80.97)	77.81	(74.50;81.12)		
Sex									0.50	0.69
Female	77.18	(73.78;80.58)	76.78	(73.29;80.27)	78.24	(74.43;82.06)	78.26	(74.47;82.05)		
Male	80.86	(77.94;83.79)	80.01	(77.12;82.91)	81.27	(78.35;84.19)	81.09	(78.21;83.97)		
Lung function at T0									0.008	0.08
≤70	77.41	(73.86;80.96)	77.07	(73.67;80.46)	77.90	(74.02;81.77)	77.91	(74.30;81.52)		
>70	80.75	(77.93;83.57)	79.81	(76.83;82.79)	81.66	(78.89;84.44)	81.40	(78.42;84.37)		
Earlier use of CFTR modulator									0.76	0.84
Yes	80.02	(77.55;82.49)	79.22	(76.70;81.74)	80.30	(77.74;82.86)	80.18	(77.61;82.74)		
No	76.33	(71.36;81.30)	76.11	(71.35;80.87)	78.52	(73.05;83.99)	78.49	(73.15;83.83)		
Use psychotropic med at T0									0.04	0.11
Yes	69.67	(61.27;78.06)	70.26	(62.24;78.27)	66.90	(58.00;75.80)	69.27	(60.65;77.90)		
No	80.16	(77.89;82.43)	79.41	(77.11;81.72)	81.20	(78.86;83.53)	80.89	(78.52;83.26)		

continued	p-values					
**Outcome**	**Post-hoc tests**					
	**T0-T1**		**T1-T2**		**T2-T3**	
	**Original**	**MI**	**Original**	**MI**	**Original**	**MI**
PedsQL Psychosocial health scores [0-100]	<0.0001	<0.0001	0.005	0.09	0.25	0.11
**Subgroups analyses**	**Post-hoc tests**					
	**Consistent difference**			**Differential change**		

	Original	MI	Original	MI
Age at T0<br><25 years<br>≥25 years	0.12	0.32	0.50	0.61
Sex<br>Female<br>Male	0.29	0.50	0.76	0.68
Lung function at T0<br>FEV_1pp ≤70<br>FEV_1pp >70	0.02	0.13	0.08	0.17
Earlier use of CFTR modulator<br>Yes<br>No	0.67	0.76	0.62	0.75
Use psychotropic medications at T0<br>Yes<br>No	0.02	0.04	0.36	0.77

	T0				T1			
	Original		MI		Original		MI	
**Outcome**	**EMM**	**(95% CI)**	**EMM**	**(95% CI)**	**EMM**	**(95% CI)**	**EMM**	**(95% CI)**
Anxiety symptoms [0-21]	3.61	(3.11;4.11)	3.64	(3.15;4.14)	3.34	(2.77;3.91)	3.45	(2.87;4.03)
**Subgroups analyses**	**EMM**	**(95% CI)**	**EMM**	**(95% CI)**	**EMM**	**(95% CI)**	**EMM**	**(95% CI)**
Age at T0								
<25 years	3.21	(2.57;3.85)	3.25	(2.63;3.88)	3.48	(2.65;4.31)	3.52	(2.70;4.35)
≥25 years	4.04	(3.27;4.82)	4.09	(3.31;4.86)	3.18	(2.41;3.95)	3.37	(2.56;4.18)
Sex								
Female	4.00	(3.21;4.79)	4.05	(3.26;4.83)	3.02	(2.23;3.81)	3.12	(2.29;3.95)
Male	3.31	(2.67;3.95)	3.33	(2.69;3.97)	3.55	(2.76;4.35)	3.72	(2.92;4.51)
Lung function at T0								
≤70	3.90	(3.19;4.61)	3.89	(3.19;4.59)	3.67	(2.83;4.52)	3.75	(2.92;4.59)
>70	3.37	(2.67;4.07)	3.45	(2.74;4.15)	3.06	(2.29;3.84)	3.21	(2.43;3.99)
Earlier use of CFTR modulator								
Yes	3.40	(2.82;3.98)	3.45	(2.88;4.03)	3.10	(2.47;3.74)	3.30	(2.64;3.95)
No	4.42	(3.45;5.38)	4.39	(3.44;5.34)	4.27	(3.00;5.54)	4.06	(2.80;5.32)
Use psychotropic med T0								
Yes	4.28	(3.20;5.36)	4.55	(3.28;5.82)	5.32	(2.94;7.69)	5.40	(3.13;7.67)
No	3.54	(3.00;4.08)	3.55	(3.03;4.08)	3.15	(2.58;3.73)	3.26	(2.67;3.85)

continued	T2				T3				p-values	
	Original		MI		Original		MI		Original	MI
**Outcome**	**EMM**	**(95% CI)**	**EMM**	**(95% CI)**	**EMM**	**overall**	**EMM**	**(95% CI)**	**overall**	**overall**
Anxiety symptoms [0-21]	3.36	(2.78;3.94)	3.39	(2.81;3.96)	3.19	(2.63;3.76)	3.35	(2.77;3.94)	0.46	0.61
**Subgroups analyses**	**EMM**	**(95% CI)**	**EMM**	**(95% CI)**	**EMM**	**(95% CI)**	**EMM**	**(95% CI)**	**Difference in visits**	
									**Original**	**MI**

Age at T0										
<25 years	3.06	(2.28;3.83)	3.08	(2.30;3.86)	3.29	(2.47;4.11)	3.44	(2.61;4.27)	0.08	0.29
≥25 years	3.66	(2.79;4.53)	3.73	(2.90;4.56)	3.11	(2.31;3.91)	3.25	(2.44;4.06)		
Sex										
Female	3.72	(2.74;4.71)	3.56	(2.63;4.48)	3.55	(2.60;4.49)	3.58	(2.64;4.51)	0.01	0.22
Male	3.13	(2.41;3.85)	3.26	(2.54;3.98)	2.96	(2.24;3.67)	3.18	(2.44;3.91)		
Lung function at T0										
≤70	3.97	(3.06;4.87)	3.90	(3.04;4.76)	3.34	(2.53;4.16)	3.39	(2.60;4.18)	0.21	0.70
>70	2.85	(2.11;3.59)	2.98	(2.23;3.73)	3.12	(2.31;3.94)	3.32	(2.49;4.16)		
Earlier use of CFTR modulator									0.23	0.38
Yes	3.16	(2.54;3.79)	3.17	(2.54;3.80)	2.91	(2.28;3.53)	3.09	(2.45;3.72)		
No	4.02	(2.59;5.45)	4.25	(2.89;5.62)	4.28	(2.99;5.58)	4.40	(3.03;5.77)		
Use psychotropic med at T0									0.02	0.26
Yes	3.84	(1.95;5.74)	3.92	(2.14;5.71)	4.44	(2.59;6.29)	4.77	(2.82;6.73)		
No	3.32	(2.71;3.94)	3.34	(2.73;3.94)	3.08	(2.48;3.68)	3.21	(2.60;3.82)		

continued	p-values					
**Outcome**	**Post-hoc tests**					
	**T0-T1**		**T1-T2**		**T2-T3**	
	**Original**	**MI**	**Original**	**MI**	**Original**	**MI**
Anxiety symptoms [0-21]	0.31	0.49	0.94	0.80	0.43	0.90

**Subgroups analyses**	**Post-hoc tests**			
	**Consistent difference**		**Differential change**	
	**Original**	**MI**	**Original**	**MI**
Age at T0<br><25 years<br>≥25 years	0.28	0.44	0.049	0.13
Sex<br>Female<br>Male	0.06	0.39	0.03	0.08
Lung function at T0<br>FEV_1pp ≤70<br>FEV_1pp >70	0.31	0.47	0.18	0.46

Earlier use of CFTR modulator<br>Yes<br>No	0.09	0.14	0.82	0.87
Use psychotropic medications at T0<br>Yes<br>No	0.01	0.12	0.25	0.43

	T0				T1			
	Original		MI		Original		MI	
**Outcome**	**EMM**	**(95% CI)**	**EMM**	**(95% CI)**	**EMM**	**(95% CI)**	**EMM**	**(95% CI)**
Depressive symptoms [0-27]	5.14	(4.52;5.75)	5.20	(4.59;5.82)	4.31	(3.67;4.95)	4.41	(3.76;5.05)
**Subgroups analyses**	**EMM**	**(95% CI)**	**EMM**	**(95% CI)**	**EMM**	**(95% CI)**	**EMM**	**(95% CI)**
Age at T0								
<25 years	4.66	(3.86;5.47)	4.78	(3.98;5.57)	4.38	(3.48;5.28)	4.44	(3.53;5.35)
≥25 years	5.65	(4.69;6.61)	5.69	(4.75;6.63)	4.21	(3.31;5.12)	4.37	(3.47;5.27)
Sex								
Female	5.56	(4.48;6.63)	5.65	(4.62;6.68)	4.60	(3.65;5.55)	4.56	(3.61;5.52)
Male	4.80	(4.06;5.55)	4.85	(4.10;5.60)	4.04	(3.19;4.89)	4.28	(3.41;5.16)
Lung function at T0								
≤70	5.97	(5.03;6.91)	5.95	(5.05;6.86)	4.77	(3.82;5.73)	4.73	(3.81;5.64)
>70	4.46	(3.65;5.27)	4.60	(3.78;5.42)	3.93	(3.07;4.79)	4.15	(3.25;5.05)
Earlier use of CFTR modulator								
Yes	4.97	(4.24;5.69)	5.06	(4.34;5.77)	4.18	(3.47;4.88)	4.34	(3.62;5.05)
No	5.77	(4.57;6.97)	5.78	(4.62;6.94)	4.78	(3.28;6.29)	4.68	(3.25;6.12)
Use psychotropic med T0								
Yes	7.22	(4.95;9.50)	7.48	(5.37;9.59)	6.32	(3.71;8.93)	6.37	(3.98;8.75)
No	4.94	(4.30;5.58)	4.98	(4.35;5.61)	4.11	(3.46;4.76)	4.21	(3.55;4.87)

<u>continued</u>	T2				T3				p-values	
	Original		MI		Original		MI		Original	MI
**Outcome**	**EMM**	**(95% CI)**	**EMM**	**(95% CI)**	**EMM**	**overall**	**EMM**	**(95% CI)**	**overall**	**overall**
Depressive symptoms [0-27]	4.06	(3.46;4.66)	4.34	(3.70;4.99)	3.89	(3.28;4.50)	3.97	(3.35;4.59)	<0.001	0.001
**Subgroups analyses**	**EMM**	**(95% CI)**	**EMM**	**(95% CI)**	**EMM**	**(95% CI)**	**EMM**	**(95% CI)**	**Difference in visits**	
									**Original**	**MI**

Age at T0										
<25 years	4.04	(3.28;4.80)	4.35	(3.47;5.24)	3.83	(2.97;4.69)	3.86	(2.99;4.73)	0.11	0.30
≥25 years	4.07	(3.13;5.01)	4.33	(3.40;5.27)	3.96	(3.08;4.84)	4.09	(3.21;4.96)		
Sex										
Female	4.52	(3.56;5.49)	4.67	(3.68;5.66)	4.38	(3.33;5.42)	4.33	(3.31;5.35)	0.14	0.54
Male	3.70	(2.93;4.46)	4.09	(3.26;4.92)	3.53	(2.78;4.28)	3.68	(2.92;4.44)		
Lung function at T0										
≤70	4.53	(3.57;5.49)	4.60	(3.64;5.56)	3.95	(3.04;4.85)	3.91	(3.05;4.78)	0.06	0.36
>70	3.68	(2.92;4.43)	4.14	(3.28;4.99)	3.91	(3.07;4.74)	4.01	(3.15;4.88)		
Earlier use of CFTR modulator									0.24	0.47
Yes	3.89	(3.21;4.58)	4.18	(3.44;4.92)	3.83	(3.17;4.48)	3.94	(3.25;4.62)		
No	4.72	(3.47;5.97)	4.98	(3.69;6.27)	4.11	(2.58;5.63)	4.09	(2.66;5.52)		
Use psychotropic med at T0									0.38	0.35
Yes	6.40	(4.27;8.52)	6.57	(4.49;8.65)	5.86	(3.90;7.83)	5.74	(3.76;7.73)		
No	3.83	(3.21;4.45)	4.12	(3.46;4.79)	3.71	(3.07;4.35)	3.79	(3.15;4.43)		

continued	p-values					
	**Post-hoc tests**					
**Outcome**	**T0-T1**		**T1-T2**		**T2-T3**	
	**Original**	**MI**	**Original**	**MI**	**Original**	**MI**
Depressive symptoms [0-27]	0.003	0.01	0.28	0.83	0.41	0.19

	**Post-hoc tests**			
**Subgroups analyses**	**Consistent difference**		**Differential change**	
	**Original**	**MI**	**Original**	**MI**
Age at T0 <25 years ≥25 years	0.16	0.55	0.17	0.30
Sex Female Male	0.04	0.24	0.94	0.78
Lung function at T0 FEV$_1$pp ≤70 FEV$_1$pp >70	0.33	0.81	0.03	0.07

Earlier use of CFTR modulator<br>Yes<br>No	0.10	0.34	0.75	0.73
Use psychotropic medications at T0<br>Yes<br>No	0.15	0.16	0.94	0.84

	T0				T1			
	Original		MI		Original		MI	
**Outcome**	**EMM**	**(95% CI)**	**EMM**	**(95% CI)**	**EMM**	**(95% CI)**	**EMM**	**(95% CI)**
Respiratory-related QoL [0-100]	68.28	(64.99;71.56)	68.78	(65.57;71.98)	89.47	(87.69;91.26)	88.65	(86.60;90.69)
**Subgroups analyses**	**EMM**	**(95% CI)**	**EMM**	**(95% CI)**	**EMM**	**(95% CI)**	**EMM**	**(95% CI)**
Age at T0								
<25 years	71.37	(66.85;75.90)	71.83	(67.45;76.20)	89.02	(86.47;91.57)	88.18	(85.32;91.05)
≥25 years	64.90	(60.17;69.64)	65.32	(60.72;69.92)	89.94	(87.39;92.49)	89.17	(86.36;91.98)
Sex								
Female	64.38	(58.98;69.79)	64.93	(59.72;70.14)	87.84	(84.70;90.99)	87.00	(83.66;90.34)
Male	71.35	(67.30;75.41)	71.80	(67.87;75.73)	90.64	(88.52;92.75)	89.94	(87.46;92.42)
Lung function at T0								
≤70	59.46	(54.63;64.30)	59.78	(55.01;64.54)	88.04	(85.42;90.66)	87.29	(84.39;90.19)
>70	75.93	(72.04;79.82)	76.03	(72.24;79.82)	90.65	(88.19;93.11)	89.74	(86.95;92.52)
Earlier use of CFTR modulator								
Yes	70.65	(67.09;74.22)	70.80	(67.30;74.30)	89.68	(87.56;91.80)	88.83	(86.52;91.15)
No	58.40	(50.48;66.32)	60.85	(53.50;68.19)	88.72	(85.65;91.80)	87.90	(84.13;91.68)
Use psychotropic med T0								
Yes	57.49	(45.98;68.99)	58.61	(47.45;69.77)	88.54	(83.44;93.64)	88.40	(82.55;94.26)
No	69.36	(65.96;72.76)	69.79	(66.48;73.09)	89.50	(87.58;91.43)	88.67	(85.51;90.83)

continued	T2				T3				p-values	
	Original		MI		Original		MI		Original	MI
**Outcome**	**EMM**	**(95% CI)**	**EMM**	**(95% CI)**	**EMM**	**overall**	**EMM**	**(95% CI)**	**overall**	**overall**
Respiratory-related QoL [0-100]	88.77	(86.44;91.10)	86.88	(84.16;89.60)	87.83	(85.37;90.29)	86.80	(84.06;89.54)	<0.001	<0.001
**Subgroups analyses**	**EMM**	**(95% CI)**	**EMM**	**(95% CI)**	**EMM**	**(95% CI)**	**EMM**	**(95% CI)**	**Difference in visits**	
									**Original**	**MI**

Age at T0										
<25 years	88.89	(85.96;91.82)	85.83	(81.95;89.72)	88.21	(85.06;91.37)	86.89	(83.11;90.66)	0.12	0.35
≥25 years	88.55	(84.86;92.24)	88.07	(84.48;91.65)	87.42	(83.56;91.29)	86.70	(82.80;90.59)		
Sex										
Female	87.42	(83.32;91.52)	85.14	(80.82;89.45)	88.08	(84.03;92.12)	86.87	(82.56;91.18)	0.01	0.15
Male	89.89	(87.19;92.59)	88.25	(84.96;91.55)	87.72	(84.53;90.90)	86.74	(83.27;90.22)		
Lung function at T0										
≤70	87.20	(83.64;90.75)	85.88	(82.02;89.73)	86.77	(83.45;90.09)	85.97	(82.35;89.59)	<0.001	<0.001
>70	89.92	(86.80;93.04)	87.69	(83.96;91.42)	88.63	(85.04;92.22)	87.46	(83.66;91.27)		
Earlier use of CFTR modulator									0.009	0.32
Yes	88.92	(86.32;91.52)	87.11	(84.05;90.16)	87.75	(84.86;90.64)	86.63	(83.48;89.79)		
No	88.36	(82.85;93.87)	86.00	(80.01;91.99)	88.17	(83.89;92.44)	87.44	(82.23;92.66)		
Use psychotropic med at T0									0.01	0.17
Yes	83.20	(76.31;90.09)	81.49	(72.91;90.07)	76.39	(65.48;87.30)	74.91	(63.57;86.26)		
No	89.25	(86.75;91.75)	87.42	(84.56;90.27)	88.96	(86.56;91.36)	87.98	(85.30;90.66)		

<u>continued</u>	**p-values**					
	**Post-hoc tests**					
**Outcome**	**T0-T1**		**T1-T2**		**T2-T3**	
	**Original**	**MI**	**Original**	**MI**	**Original**	**MI**
Respiratory-related QoL [0-100]	<0.001	<0.001	0.56	0.24	0.38	0.96

**Subgroups analyses**	**Post-hoc tests**			
	**Consistent difference**		**Differential change**	
	**Original**	**MI**	**Original**	**MI**
Age at T0 <25 years ≥25 years	0.20	0.94	0.14	0.05
Sex Female Male	0.01	0.17	0.18	0.23
Lung function at T0 FEV_1pp ≤70 FEV_1pp >70	0.09	0.11	<0.001	<0.001

Earlier use of CFTR modulator<br>Yes<br>No	0.06	0.44	0.02	0.10
Use psychotropic medications at T0<br>Yes<br>No	0.03	0.22	0.06	0.09

Supplement 5'

	p-values							
	Visit-specific contrasts							
**Subgroups analyses on psychosocial health**	**T0**		**T1**		**T2**		**T3**	
	**Original**	**MI**	**Original**	**MI**	**Original**	**MI**	**Original**	**MI**
Age at T0<br><25 years<br>≥25 years	0.04	0.04	0.16	0.27	0.02	0.06	0.05	0.10
Sex<br>Female<br>Male	0.16	0.17	0.31	0.49	0.11	0.16	0.21	0.24
Lung function at T0<br>$FEV_1pp \leq 70$<br>$FEV_1pp > 70$	0.006	0.006	0.37	0.30	0.15	0.23	0.12	0.14
Earlier use of CFTR modulator<br>Yes<br>No	0.24	0.19	0.39	0.48	0.19	0.26	0.56	0.59
Use psychotropic medications at T0<br>Yes<br>No	0.008	0.004	0.01	0.01	0.02	0.03	0.004	0.01

continued	p-values							
**Subgroups analyses on anxiety symptoms**	**Visit-specific contrasts**							
	**T0**		**T1**		**T2**		**T3**	
	**Original**	**MI**	**Original**	**MI**	**Original**	**MI**	**Original**	**MI**
Age at T0<br><25 years<br>≥25 years	0.10	0.10	0.60	0.80	0.30	0.26	0.75	0.74
Sex<br>Female<br>Male	0.18	0.16	0.35	0.30	0.34	0.61	0.32	0.50
Lung function at T0<br>FEV_1pp ≤70<br>FEV_1pp >70	0.29	0.38	0.29	0.34	0.06	0.11	0.70	0.91
Earlier use of CFTR modulator<br>Yes<br>No	0.07	0.10	0.10	0.29	0.27	0.16	0.06	0.09
Use psychotropic medications at T0<br>Yes<br>No	0.22	0.16	0.08	0.08	0.59	0.54	0.15	0.13

continued	p-values							
	Visit-specific contrasts							
**Subgroups analyses on depressive symptoms**	**T0**		**T1**		**T2**		**T3**	
	**Original**	**MI**	**Original**	**MI**	**Original**	**MI**	**Original**	**MI**
Age at T0<br><25 years<br>≥25 years	0.12	0.15	0.80	0.92	0.96	0.97	0.83	0.72
Sex<br>Female<br>Male	0.25	0.22	0.39	0.67	0.18	0.37	0.19	0.31
Lung function at T0<br>$FEV_1pp \leq 70$<br>$FEV_1pp > 70$	0.02	0.03	0.20	0.38	0.17	0.48	0.95	0.87
Earlier use of CFTR modulator<br>Yes<br>No	0.26	0.30	0.47	0.67	0.25	0.29	0.74	0.85
Use psychotropic medications at T0<br>Yes<br>No	0.05	0.03	0.10	0.09	0.02	0.03	0.04	0.07

continued	p-values							
**Subgroups analyses on respiratory-related QoL**	**Visit-specific contrasts**							
	**T0**		**T1**		**T2**		**T3**	
	**Original**	**MI**	**Original**	**MI**	**Original**	**MI**	**Original**	**MI**
Age at T0								
<25 years	0.05	0.04	0.61	0.62	0.88	0.40	0.75	0.95
≥25 years								
Sex								
Female	0.04	0.04	0.14	0.16	0.32	0.25	0.89	0.96
Male								
Lung function at T0								
FEV_1pp ≤70	<0.0001	<0.0001	0.15	0.23	0.25	0.50	0.45	0.56
FEV_1pp >70								
Earlier use of CFTR modulator								
Yes	0.006	0.02	0.61	0.67	0.85	0.75	0.87	0.80
No								
Use psychotropic medications at T0								
Yes	0.05	0.06	0.71	0.93	0.10	0.20	0.03	0.03
No								

'Table 3' in the main text.

	Original	MI	Original	MI	Original		MI		Original	MI
**Clinical parameters**	**T0, EMM**	**T0, EMM**	**T2, EMM**	**T2, EMM**	**Difference**	**(95% CI)**	**Difference**	**(95% CI)**	**p-value**	**p-value**
BMI (kg/m^2)	21.12	21.12	21.79	21.77	0.67	(0.48; 0.85)	0.65	(0.44;0.86)	<0.0001	<0.0001
FEV_1pp	73.18	73.18	86.27	85.69	13.10	(11.39;14.81)	12.51	(10.51;14.51)	<0.0001	<0.0001
SCC ($mmol \cdot L^{-1}$)	84.27	84.08	34.93	34.08	-49.34	(-53.03; -45.64)	-50.00	(-54.19;-45.81)	<0.0001	<0.0001
