# Supplementary material for: Mental Health Changes in Adolescents and Adults With Cystic Fibrosis After Initiation of Elexacaftor/Tezacaftor/Ivacaftor Therapy: Insights From the Longitudinal Resilience Impacted by Positive Stressful Events (RISE) Study
Source: CHEST Pulm. 2025 Feb 7;3(3):100146. doi: 10.1016/j.chpulm.2025.100146 (PMC13418347; doi:10.1016/j.chpulm.2025.100146)
Supplement: e-Online Data [file mmc3.pdf]

Supplement 3 - ***response rate regarding the RISE-questionnaires***

Questionnaires, total participants n=177				
**Time points**	**T0**	**T1**	**T2**	**T3**
**Completed**	174 (98.31)	146 (82.49)	141 (79.66)	142 (80.23)
100% completed	158 (89.27)	140 (79.10)	137 (77.40)	139 (78.53)
Partially completed	16 (9.04)	6 (3.39)	4 (2.26)	3 (1.69)
Not completed	3 (1.69)	31 (17.51)	36 (20.34)	35 (19.77)

Data are presented as n, or n (%).
