# Supplementary material for: Mental Health Changes in Adolescents and Adults With Cystic Fibrosis After Initiation of Elexacaftor/Tezacaftor/Ivacaftor Therapy: Insights From the Longitudinal Resilience Impacted by Positive Stressful Events (RISE) Study
Source: CHEST Pulm. 2025 Feb 7;3(3):100146. doi: 10.1016/j.chpulm.2025.100146 (PMC13418347; doi:10.1016/j.chpulm.2025.100146)
Supplement: e-Online Data [file mmc4.pdf]

# Supplement 4: Visualization of subgroup analysis

**Figure 2A - PedsQL Psychosocial health scores based on subgroups**

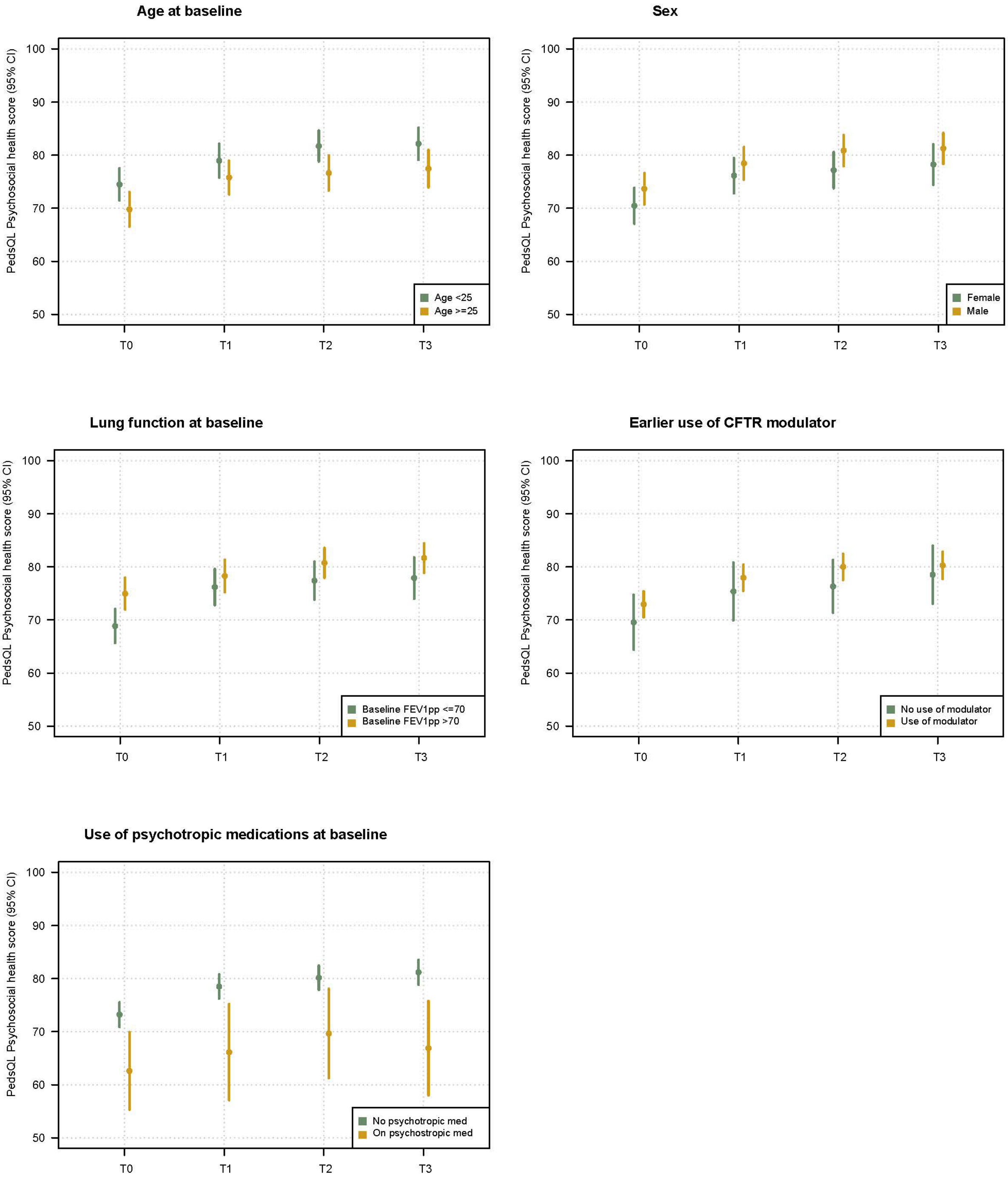

**Figure 2B - GAD-7 scores based on subgroups**

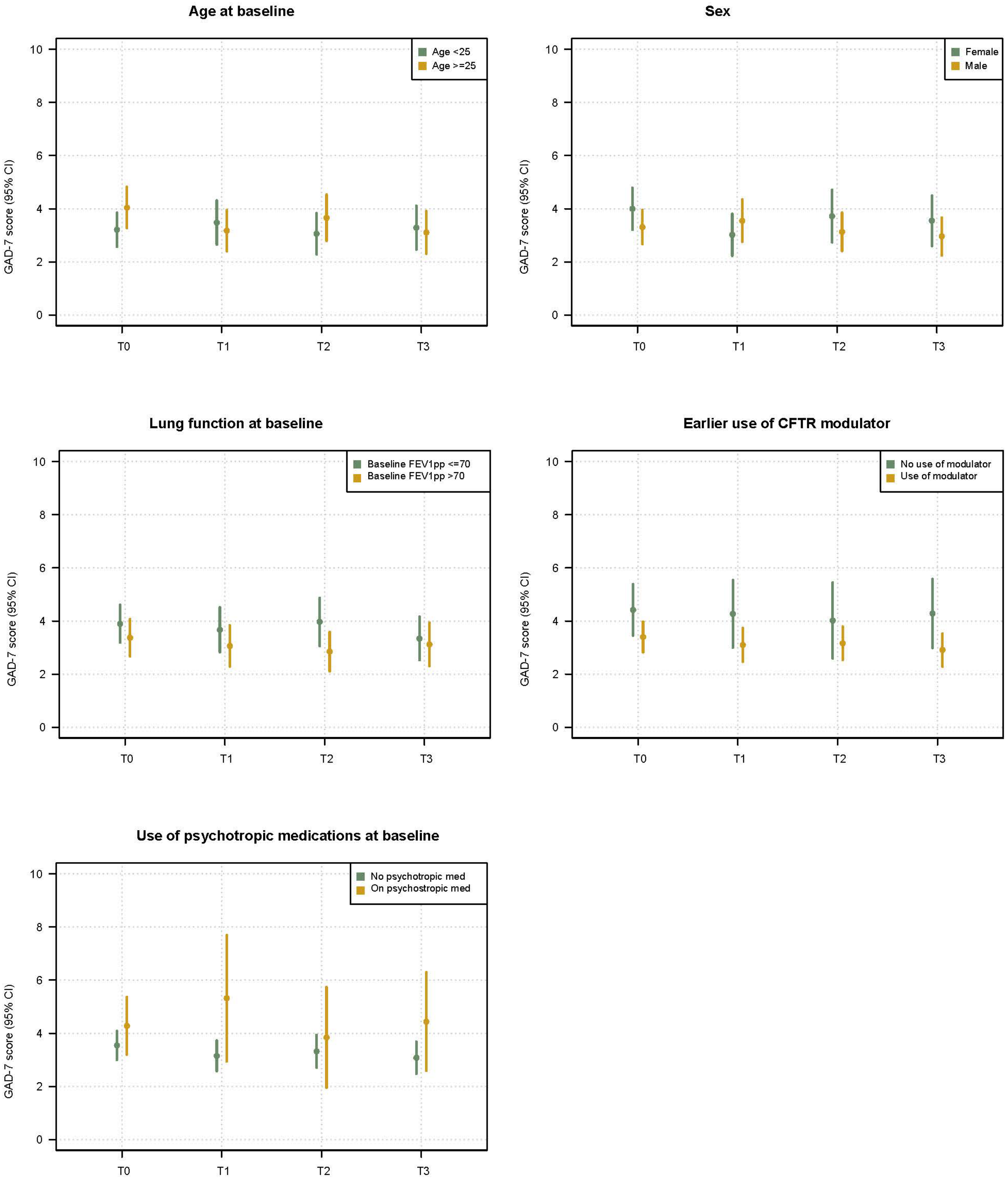

Figure 2C - PHQ-9 scores based on subgroups

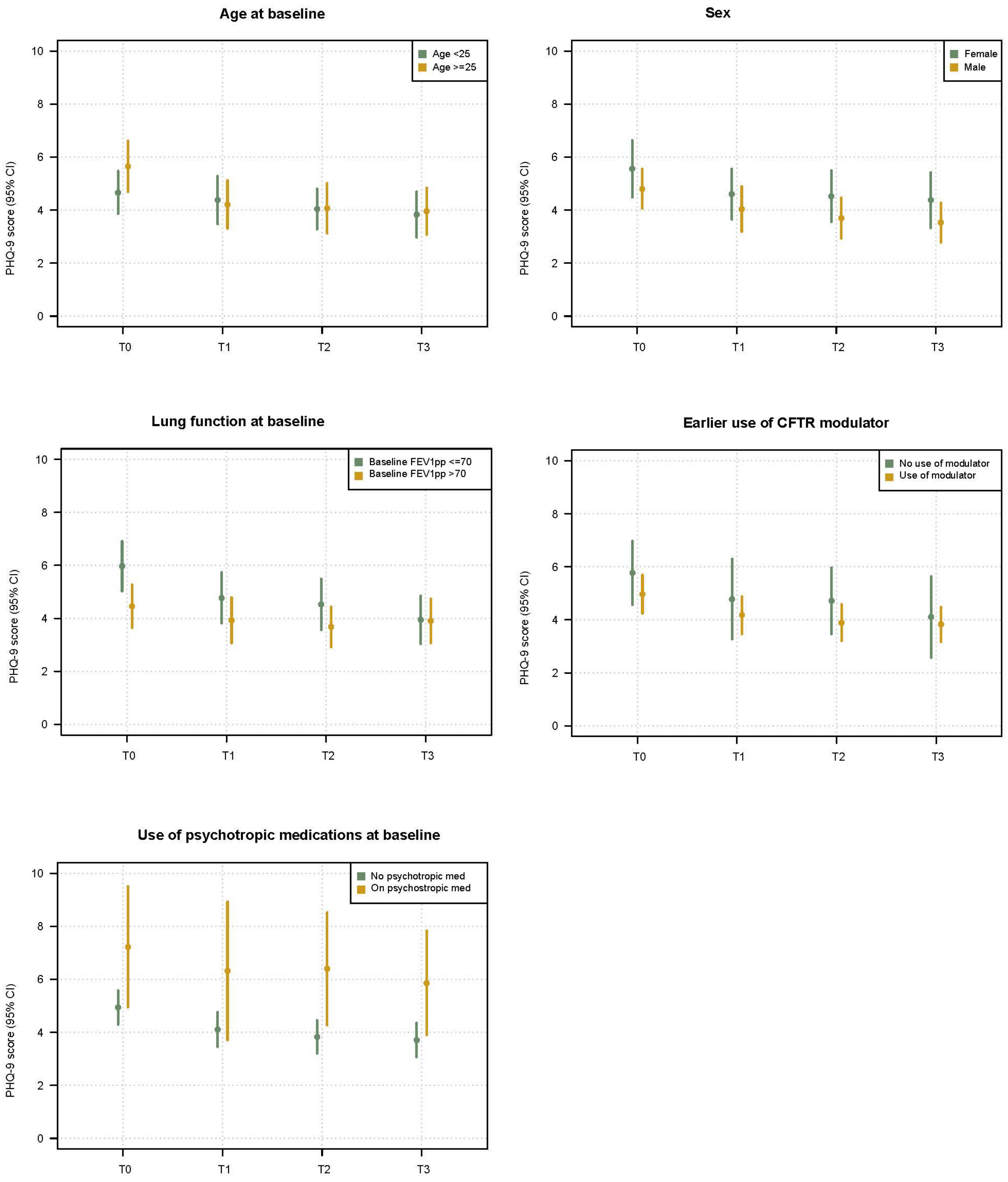

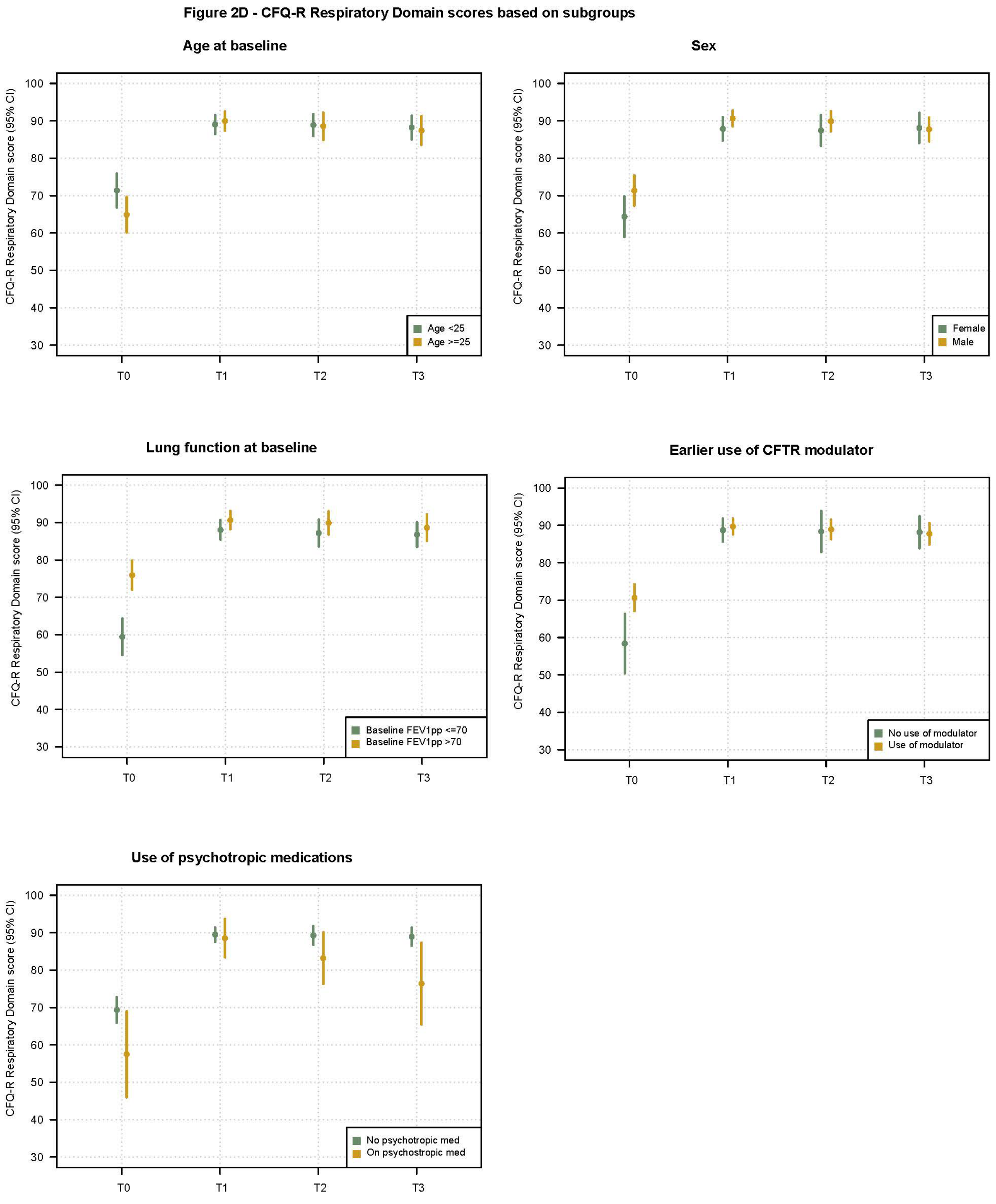


**Figure 2**: Estimated marginal means with 95% confidence intervals at T0, T1, T2 and T3 with A. PedsQL psychosocial health scores; B. GAD-7 scores; C. PHQ-9 scores; and D. CFQ-R respiratory domain scores. Subgroups based at age at T0, sex, earlier use of CFTR-modulator, $FEV_1$pp at T0, and use of psychotropic medication at T0. Med = medications Original data is used.
