# Supplementary material for: Mental Health Changes in Adolescents and Adults With Cystic Fibrosis After Initiation of Elexacaftor/Tezacaftor/Ivacaftor Therapy: Insights From the Longitudinal Resilience Impacted by Positive Stressful Events (RISE) Study
Source: CHEST Pulm. 2025 Feb 7;3(3):100146. doi: 10.1016/j.chpulm.2025.100146 (PMC13418347; doi:10.1016/j.chpulm.2025.100146)
Supplement: e-Online Data [file mmc5.pdf]

Supplement 5 – Visit-specific contrasts

	p-values			
**Outcome: Psychosocial health**	**Visit-specific contrasts**			
	**T0**	**T1**	**T2**	**T3**
Age at T0<br><25 years<br>≥25 years	0.04	0.16	0.02	0.05
Sex<br>Female<br>Male	0.16	0.31	0.11	0.21
Lung function at T0<br>FEV_1pp ≤70%<br>FEV_1pp >70%	0.006	0.37	0.15	0.12
Earlier use of CFTR modulator<br>Yes<br>No	0.24	0.39	0.19	0.56
Use psychotropic medications at T0<br>Yes<br>No	0.008	0.01	0.02	0.004

	p-values			
**Outcome: Anxiety symptoms**	**Visit-specific contrasts**			
	**T0**	**T1**	**T2**	**T3**
Age at T0<br><25 years<br>≥25 years	0.10	0.60	0.30	0.75
Sex<br>Female<br>Male	0.18	0.35	0.34	0.32
Lung function at T0<br>FEV_1pp ≤70%<br>FEV_1pp >70%	0.29	0.29	0.06	0.70
Earlier use of CFTR modulator<br>Yes<br>No	0.07	0.10	0.27	0.06
Use psychotropic medications at T0<br>Yes<br>No	0.22	0.08	0.59	0.15

	p-values			
**Outcome: Depressive symptoms**	**Visit-specific contrasts**			
	**T0**	**T1**	**T2**	**T3**
Age at T0<br><25 years<br>≥25 years	0.12	0.80	0.96	0.83
Sex<br>Female<br>Male	0.25	0.39	0.18	0.19
Lung function at T0<br>FEV_1pp ≤70%<br>FEV_1pp >70%	0.02	0.20	0.17	0.95
Earlier use of CFTR modulator<br>Yes<br>No	0.26	0.47	0.25	0.74

Use psychotropic medications at T0<br>Yes<br>No	0.05	0.10	0.02	0.04

	**p-values**			
**Outcome: Respiratory-related QoL**	**Visit-specific contrasts**			
	**T0**	**T1**	**T2**	**T3**
Age at T0<br><25 years<br>≥25 years	0.05	0.61	0.88	0.75
Sex<br>Female<br>Male	0.04	0.14	0.32	0.89
Lung function at T0<br>FEV_1pp ≤70%<br>FEV_1pp >70%	<0.0001	0.15	0.25	0.45
Earlier use of CFTR modulator<br>Yes<br>No	0.006	0.61	0.85	0.87
Use psychotropic medications at T0<br>Yes<br>No	0.05	0.71	0.10	0.03
