# Supplementary material for: Mental Health Changes in Adolescents and Adults With Cystic Fibrosis After Initiation of Elexacaftor/Tezacaftor/Ivacaftor Therapy: Insights From the Longitudinal Resilience Impacted by Positive Stressful Events (RISE) Study
Source: CHEST Pulm. 2025 Feb 7;3(3):100146. doi: 10.1016/j.chpulm.2025.100146 (PMC13418347; doi:10.1016/j.chpulm.2025.100146)
Supplement: e-Online Data [file mmc6.pdf]

Supplement 6 – ***Clinical changes after ETI therapy***

Of the 177 people at T0, 151 (85.31%) participants attended the medical consultation on T2. Only 74 participants had their sweat chloride concentration measured at T2 due to a lack of sweat chloride tests (an international event beyond the control of the study team).

A majority of participants who attended the medical evaluation at T2 (78.81%, n=119 of 151) experienced side effects from ETI within six months of initiation, with some involving mental health-related changes. To start with the mental health-related changes, these were noted both from an open-ended question and by verbally reporting a predefined list of side effects. In total, 21.85% of the participants experienced fatigue (n=26 of 119), 10.92% experienced mood changes, 5.04% experienced sleep problems, and 2.52% of the participants experienced concentration problems, were restless or forgetful. Moreover, some participants had changes in their psychotropic medication use; sixteen used these medications at T0 and twelve at T2. Of these twelve participants, ten already previously used psychotropic medication, while two started taking medication during the study."

Also physical side effects were noted. Of the 119 participants, 48 (40.34%) participants reported coughing, 69 (57.98%) sputum increase, 30 (25.21%) headache, 18 (15.13%) diarrhea, 27 (22.69%) abdominal pain, 3 (2.52%) hemoptysis, 14 (11.76%) rhinorrhea, 6 (5.04%) hoarseness, 7 (5.88%) sore throat, and 20 participants reported (16.81%) rash.. Side effects reported in open answer format were: obstipation (n=7 5.88%), dry eyes or other eye related symptoms (n=6, 5.04%), being dizzy (n=5, 4.20%), tinnitus (n=5, 4.20%), skin-related problems (n=4, 3.36%), cold-like symptoms (n=3, 2.52%), different sputum production (n=3, 2.52%), fever (n=3, 2.52%), liver function problems (n=3, 2.52%), gall or kidney stones (n=2, 1.68%), nausea (n=2, 1.68%), shortness of breath (n=2, 1.68%), weight gain (n=2, 1.68%), diaphragmatic pain (n=1, 0.84%), hypertension (n=1, 0.84%), having more energy (n=1, 0.84%), headache (n=1, 0.84%), hot flash (n=1, 0.84%), more hungry (n=1, 0.84%), more often getting stung by mosquitoes (n=1, 0.84%), and pain in testicles (n=1, 0.84%). At T2, 24 (20.16%) participants were still experiencing one side effect, twelve (10.1%) participants experienced two side effects, five (4.20%) participants experienced three side effects, and two (1.68%) participants experienced four side effects at T2. We do not have any data on side effects at T1 and T3.
