# Supplementary material for: Mental Health Changes in Adolescents and Adults With Cystic Fibrosis After Initiation of Elexacaftor/Tezacaftor/Ivacaftor Therapy: Insights From the Longitudinal Resilience Impacted by Positive Stressful Events (RISE) Study
Source: CHEST Pulm. 2025 Feb 7;3(3):100146. doi: 10.1016/j.chpulm.2025.100146 (PMC13418347; doi:10.1016/j.chpulm.2025.100146)
Supplement: e-Online Data [file mmc7.pdf]

**Supplement 7**

Comparison of baseline distributions on age, lung function, PedsQL, PHQ-9, and CFQ-R RD, GAD-7 scores between subjects who did and did not complete the RISE surveys at T3 indicated potential differences in CFQ-R RD and GAD-7 (Supplement 7). We aimed to mitigate potential bias by using multiple imputation techniques. The EMMs estimated using the imputed data were similar to the EMMs estimated without imputation, and therefore, these data suggest limited attrition bias.

| Completing RISE questionnaires at T3 | Variable at baseline | Mean | (SD) | Median | (interquartile range) |
|---|---|---|---|---|---|
| No | Age | 23.87 | 8.04 | 21.85 | (17.78; 29.39) |
| No | FEV1pp | 75.29 | 16.74 | 76.50 | (66.75; 87.50) |
| No | PedsQL psychosocial health score | 72.45 | 14.91 | 74.17 | (62.92; 85.00) |
| No | GAD-7 score | 2.71 | 2.90 | 2.00 | (1.00; 4.50) |
| No | PHQ-9 score | 5.10 | 4.04 | 4.00 | (2.00; 8.50) |
| No | CFQ-R RD score | 72.42 | 21.78 | 83.33 | (66.67; 88.89) |
| Yes | Age | 26.32 | 8.93 | 25.33 | (19.30; 31.45) |
| Yes | FEV1pp | 72.74 | 20.32 | 73.50 | (57.25; 89.00) |
| Yes | PedsQL psychosocial health score | 72.48 | 14.74 | 73.33 | (61.67; 83.33) |
| Yes | GAD-7 score | 3.78 | 3.36 | 3.00 | (1.00; 6.00) |
| Yes | PHQ-9 score | 5.09 | 4.08 | 4.00 | (2.00; 7.00) |
| Yes | CFQ-R RD score | 68.07 | 20.68 | 66.67 | (55.56; 83.33) |

CFQ-R RD = cystic fibrosis questionnaire revised respiratory domain
GAD-7 = Generalized Anxiety Disorder-7
PedsQL 4.0 = Pediatric Quality of Life Inventory 4.0
PHQ-9 = Patient Health Questionnaire-9
